# Supplementary material for: Sociodemographic Associations and COVID-19 Symptoms Following One Year of Molecular Screening for SARS-CoV-2 Among Healthcare Workers
Source: Viruses. 2025 Dec 16;17(12):1622. doi: 10.3390/v17121622 (PMC12737519; doi:10.3390/v17121622)
Supplement: Supplementary file 1 [file viruses-17-01622-s001.zip › viruses-3927693-supplementary.pdf]

Supplementary Table S1 - Model Coefficients – Prior COVID-19

Predictor	Estimate	Std. Error	Z	p	Odds Ratio	95% CI (Lower – Upper)
Intercept	0.3887	1.0625	0.3658	0.714	1.4750	0.1838 – 11.8353
Age	-0.0034	0.0204	-0.1681	0.866	0.9966	0.9574 – 1.0373

Note. Estimates represent log-odds of "Prior COVID-19 = YES" vs. "NO".

Supplementary Table S2 - Model Coefficients – One COVID-19 infection during the study

Predictor	Estimate	Std. Error	Z	p	Odds Ratio	95% CI (Lower – Upper)
Intercept	1.1586	1.2168	0.9522	0.341	3.1854	0.2934-34.5849
Age	-0.0356	0.0239	-1.4904	0.136	0.9650	0.9209-1.0113

Note. Estimates represent log-odds of "One COVID-19 infection = YES" vs. "NO".
